# Bacterial memory in antibiotic resistance evolution and nanotechnology in evolutionary biology

**DOI:** 10.1016/j.isci.2023.107433

**Published:** 2023-07-20

**Authors:** Chengdong Zhang, Yan Kong, Qingxin Xiang, Yayun Ma, Quanyi Guo

**Affiliations:** 1 School of Environment, Beijing Normal University, Beijing 100875, China

**Keywords:** Toxicology, Microbiology, Nanomaterials

## Abstract

Bacterial memory refers to the phenomenon in which past experiences influence current behaviors in response to changing environments. It serves as a crucial process that enables adaptation and evolution. We first summarize the state-of-art approaches regarding history-dependent behaviors that impact growth dynamics and underlying mechanisms. Then, the phenotypic and genotypic origins of memory and how encoded memory modulates drug tolerance/resistance are reviewed. We also provide a summary of possible memory effects induced by antimicrobial nanoparticles. The regulatory networks and genetic underpinnings responsible for memory building partially overlap with nanoparticle and drug exposures, which may raise concerns about the impact of nanotechnology on adaptation. Finally, we provide a perspective on the use of nanotechnology to harness bacterial memory based on its unique mode of actions on information processing and transmission in bacteria. Exploring bacterial memory mechanisms provides valuable insights into acclimation, evolution, and the potential applications of nanotechnology in harnessing memory.

## Graphical abstract

**Figure:**
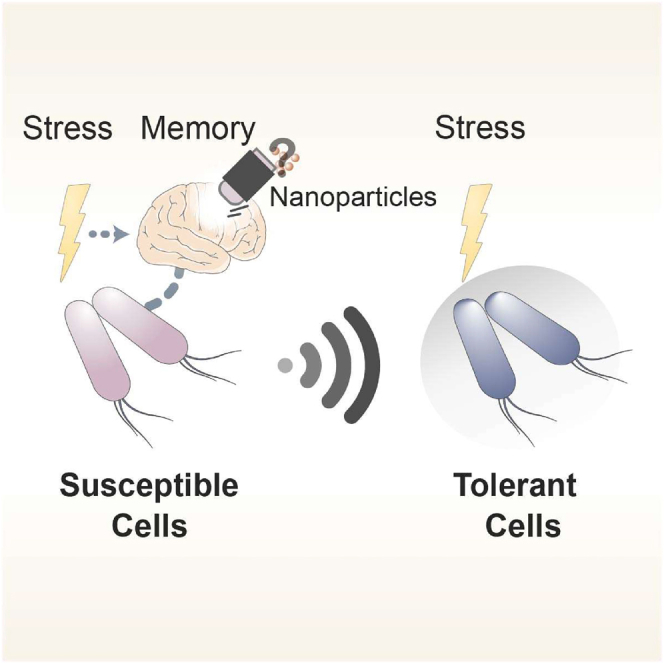

## Introduction

The term “bacterial memory” is used to describe the phenomena by which bacterial cells are able to respond to their current conditions based on prior experience. This history-dependent behavior is essential for bacteria to adapt quickly and survive in fluctuating environments. Cellular memory can also trigger bet-hedging at the population level, giving rise to a phenotypically heterogeneous landscape. Bet-hedging is an evolutionary strategy to diversify phenotypes, resulting in a selective advantage that optimizes survival under fluctuating conditions.^1^ From the perspective of bacterial evolution, the founding of induced memory responses (i.e., memorization of cellular responses to historical situations and transmission of information within/between generations) adds a time dimension that may bridge the gap between short-term induced individual responses and long-term evolutionary genotypic adaptation.

In particular, bacterial memory plays an imperative role in the development of antibiotic tolerance/resistance, which is now one of the most challenging problems in human health.^2^^,^^3^^,^^4^ For example, cells exhibit diverse degrees of tolerance to antibiotics when previously exposed to various environmental cues, namely, acid,^5^ heat/cold,^6^^,^^7^^,^^8^ salt,^6^ starvation,^5^^,^^6^^,^^7^^,^^8^^,^^9^ and oxidative stress.^5^^,^^6^^,^^7^ These stress-triggered responses, e.g., heat/cold shock response,^6^^,^^7^ string response,^5^^,^^7^ SOS response,^6^^,^^7^^,^^9^ and envelope stress response,^7^^,^^10^ are also involved in the mechanisms responsible for drug tolerance/resistance. For example, the envelope stress response is initiated to maintain envelope homeostasis, repair damage, and ensure cell integrity.^11^ The induced envelope stress response subsequently mediates *Escherichia coli* susceptibility to tobramycin while promoting aminoglycoside resistance in *Pseudomonas aeruginosa*.^10^ There have been a series of reviews^5^^,^^6^^,^^7^^,^^8^^,^^9^ on the relationship between the bacterial response to environmental stresses and the subsequent development of drug tolerance; therefore, we will not include this information in this review. Similarly, one-time exposure to disinfectants or sublethal antibiotics triggers multifaceted damage repair and stress-mediated metabolism, resulting in tolerance to drug therapies and the rapid evolution of resistance.^12^ Notably, in the current study, we distinguish between adaptive responses and memory effects in terms of timescale. In general, bacterial memory lasts from a few seconds to a few generations, while bacterial adaptation is assessed after serial passaging over periods ranging from days to months. Nevertheless, there are still controversial issues regarding the relationships between history-related behaviors and poststress effects, lag time before regrowth, heterogeneous population, protein aggregation, epigenetic alteration, biofilm and collective memory, etc. It remains a priority to disentangle the essential mechanisms for the recruitment of historical information and to determine how the carry-over effects assist in the survival of bacterial populations during antibiotic treatment. More concerns are emerging about the role that microbial memory plays in microbial evolution and clinical microbiology. Genetic background drift and transcriptional programming may persist after the transient stimulus, which can be carried between generations and direct the evolution of adaptations and facilitate the emergence of resistance.^13^

Nanotechnology has recently received considerable attention due to its versatile biocidal mechanisms and tunable properties and has been recognized as an effective weapon against multidrug-resistant bacteria in the postantibiotic era. The material sciences of antimicrobial nanoparticles,^14^^,^^15^ biocidal mechanisms,^14^ and potential applications as delivery carriers,^14^^,^^15^ light-sensitive materials^14^ (for photokilling), and adjuvants^15^ have been previously reviewed. Nevertheless, it is largely unknown whether bacteria have a memory for exposure to nanoparticles. Is the encoding regulation of memory similar or dissimilar to that of past experiences with drugs? How is this historical information relevant to drug tolerance/resistance? Understanding the consistent gene expression patterns that occur after nanoparticle exposure (referred to as nanomaterial-induced memory) can provide valuable insights into the implications for drug tolerance/resistance. By unraveling the nano-bio interactions and the connections of proteins that persist after exposure, we can gain a better understanding of the potential applications of nanotechnology to reduce drug tolerance. Furthermore, gaining a comprehensive understanding of the regulatory circuits associated with historical nano-exposure can highlight the promise of nanotechnology in harnessing bacterial memory and minimizing the evolution of resistance.

In this review, we first summarize memory-related behaviors and underlying mechanisms in the context of transcriptional memory, hysteresis memory, and response memory. These memory responses can be categorized based on the mode of information transmission, specifically within a generation, during the regrowth process, and contingent upon the recurrent inducer. We then recapitulate the link between phenotype and genotype transition/evolution stemming from history-dependent responses. We further briefly describe how the memory response influences bacterial susceptibility to antibiotics. In particular, we review studies involving nanoparticle exposure-induced memory and propose potential applications of nanotechnology for intervention, considering the double-edged impacts. Finally, the remaining questions and future perspectives are discussed.

## Transcriptional memory, hysteresis memory, and response memory

**Box 1. Definitions**

• Bet-hedging: Bet-hedging is an evolutionary strategy to diversify phenotypes, resulting in a selective advantage that optimizes survival under fluctuating conditions.
• Stringent response: The stringent response is a conserved stress response that is activated primarily by nutrient starvation.
• SOS response: The SOS response is a general response to DNA damage that initiates DNA repair and induces mutagenesis.
• Envelop stress response: The envelope stress response is initiated as a means to maintain envelope homeostasis, repair any damage, and ensure the overall integrity of the cellular envelope.
• Antibiotic tolerance: Bacteria can survive high doses of antibiotics without difference in the minimal inhibition concentration (MIC) relative to susceptible ones.
• Antibiotic resistance: Bacteria can survive high doses of drugs with elevated MIC values.
• Poststress effects: The poststress effect refers to the phenomenon of continuous bacterial killing even after the complete removal of antibiotics or stressors. This effect is mainly attributed to the formation of secondary reactive oxygen species (ROS).
• Lag time: The lag time describes an interval of adaptation after which the bacteria re-enter a logarithmic growth period.
• Persisters: Persisters are a subpopulation of nongrowing bacteria capable of tolerating multiple stresses.
• Epigenetic alteration: Epigenetic alteration refers to the modification of the chemical structure of DNA without changing the underlying DNA coding sequence.
• Heteroresistance: Bacterial resistance is the condition in which only a subset of cells within a population are resistant.
• Epistasis: Epistasis refers to the phenomenon where the impact of a mutation is influenced by the specific genetic background or context in which it occurs.
• Chaperon-like activity: Chaperone-like activity is characterized by the capability to aid in the folding, unfolding, and remodeling of large proteins.

### Transcriptional memory

**Figure 1:**
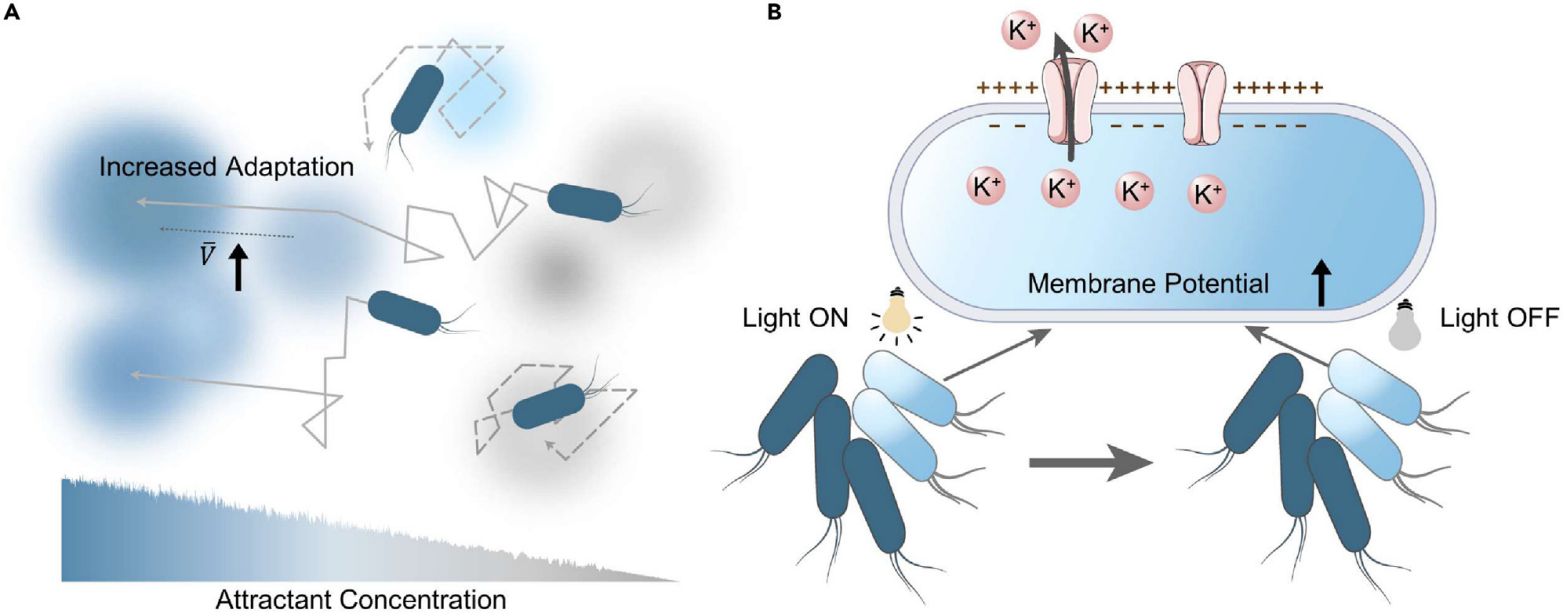
Examples of transcriptional memory (A) Bacteria exhibit an increased adaptive response speed based on information gathered from previous experience.^16^ (B) Light changes the bacterial membrane potential, and the changes are preserved even when the light is switched off.^18^ (A and B) Reproduced under the terms of the CC-BY Creative Commons Attribution 4.0 International License (http://creativecommons.org/licenses/by/4.0/). Copyright 2020.

Transcriptional memory (short memory within a generation) refers to the phenomena by which individual cells previously exposed to stimulus have faster recruitment of transcription factors to genes upon restimulation. For example, bacteria employ short-range memory to direct chemotaxis behavior based on collected information (Figure 1A).^16^ It has been shown that *E*. *coli* cells that have previously navigated rugged chemoattractant landscapes are able to reduce their adaptation time and thus enhance their subsequent drift velocity by memorizing information from environmental correlations. Maximal advantage is observed when the memory is comparable to the timescale of the fluctuations sensed during the preceding swim. It has been suggested that bacteria optimize their memory by regulating the dynamics of receptor methylation, varying not only the level but also the order of methylation sites.^17^ In particular, cells with short memories of motility could produce heterogeneous subpopulations of varied responses, inducing relatively long-lived bet-hedging at the population level (from seconds to minutes). The ability of bacteria to actively navigate their microenvironment using chemotaxis can greatly affect their nutrient uptake, flagella-related functions, and adaptation. Thus, memory regulation in chemotaxis may be a prospective target for modulating drug tolerance.

Another fascinating short-term memory-based feature is that transient optical perturbations generate potassium channel-mediated modifications in the membrane potential of *Bacillus subtilis* within the biofilm (Figure 1B).^18^ These exposed cells respond differently to the extracellular osmotic environment compared to unexposed cells, which can carry on for hours after the temporary optical stimulus. It is widely believed that membrane voltages potentiate drug uptake, and bacteria that persist with depolarizing membrane potentials are more resistant to multiple antibiotics.^19^ In general, these transition memories can be attributed to modulation of overall mRNA turnover and to the persistence of specific gene regulators that favor responses to changing environments. Evidently, these transient memories comprise a scenario of long-term stimulus-response selection that may further direct both phenotypic and genotypic evolution at both the single-cell and community levels.

### Hysteresis memory

**Figure 2:**
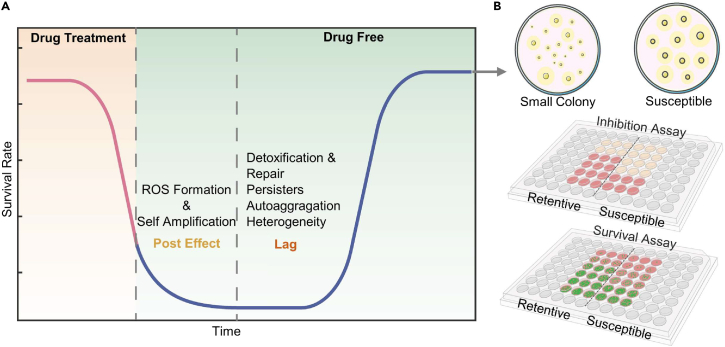
Relationship between drug treatment, growth hysterisis, and the response after regrowth (Aand B) (A) Mechanisms for hysteresis memory during growth dynamics and (B) memory-related responses after regrowth.

The timing of regrowth (Figure 2A) following a previous exposure is defined as one of the characteristics of hysteresis memory or history-dependent recovery, and the underlying mechanisms are reviewed in the context of the poststress effect, detoxification and repair mechanisms, persister formation, bacterial aggregation, and the evolution of phenotypic heterogeneity at the population level.

The poststress effect refers to continuous bacterial killing after the complete removal of antibiotics,^20^^,^^21^ which is mostly ascribed to secondary ROS formation independent of drug type.^22^ Hong et al. discovered that *E*. *coli* exposed to multiple lethal antibiotics did not die during treatment but instead died after incubation on drug-free agar due to continuous ROS production.^23^ Resuscitation, therefore, requires an additional step; namely, overcoming ROS-mediated secondary damage. Bacteria use hysteresis to necessitate detoxification and repair processes after preexposure, e.g., the dissociation of residual drugs from targets, the efflux of drugs outside the cell, the repair of DNA damage, and the synthesis of RNA, ribosomes, and essential enzymes for regrowth.^20^ As an example, proteomic analysis revealed that functional genes involved in nucleotide biosynthesis, gene transcription, protein translation, and coenzyme biosynthesis were highly differentially expressed in *Lactarius lactis* during the lag phase.^24^ The relevance of the lag time to all these dynamic processes was previously reviewed in detail.^20^^,^^25^

Furthermore, persisters are considered an important subpopulation responsible for the lag because they enter a metabolically inactivate status (non-multiplying) after stimulus. Persisters are induced via the SOS response and the toxin-antitoxin system in response to DNA damage and are modulated by the growth factor guanosine tetraphosphate (ppGpp).^9^ These persister phenotypes can retain memories for days to weeks before resuscitation.^26^ An interesting study demonstrated the memory effect at the single-cell level, and *E*. *coli* cells that entered dormancy last were also the first to resume growth in response to nutrients, following a “last in, first out” rule.^27^ Unlike slow-growing dying cells, these cells maintained high activity in efflux, transcription-coupled DNA repair, and protein disentanglement.^28^ As an example, Pu et al. revealed that the ability of persisters to employ functional *DnaK-ClpB* machinery, which expedites protein disaggregation in an ATP-dependent mode, governs the lag time for bacterial regrowth.^29^

Hysteresis memory is also partially ascribed to bacterial autoaggregation at the population level, which is mediated by self-identifying surface structures such as proteins and exopolysaccharides or by altruistic programmed cell death.^30^^,^^31^ Autoaggregation is typically triggered by oxidative stress (such as the previously mentioned poststress effect), and this survival tactic allows the entire population to tolerate the antibiotic. For example, for *P*. *aeruginosa*, depletion aggregation was induced by mucin/extracellular DNA due to entropic forces between uncharged or electrically charged polymers and cells, which subsequently survived high doses of ciprofloxacin.^32^ Notably, self-aggregation may or may not be associated with biofilm formation.^33^ Thus, hysteresis is required for attached bacteria to escape from self-adjoining surface protein interactions, presumably mediated by van der Waals forces. Finally, while encountering new resources, the wide lag distribution indicates that isogenic individuals develop phenotypic heterogeneity over the lag time, exhibiting a growth versus stress trade-off at the population level. Lag, often described as a preparatory phase, is indeed a dynamic, adaptive, and evolvable period that exhibits the characteristic features of metal ion accumulation, time-dependent membrane potential shift, nutrient sensing, and the emergence of diverse adaptive phenotypes.^34^ Overall, bacteria retain a memory of the total duration of previous exposures, and a mathematic investigation of the lag time distribution enables the prediction of survival upon subsequent treatment.^35^

### Memory response

The memory response (Figure 2B) refers to distinct morphological changes after regrowth, reduced growth inhibition, and higher survival upon subsequent treatment when compared to unexposed bacteria.

Small colony formation has been suggested as one of the typical morphological variants of poststress regrowth. For instance, Vulin et al. showed that a prolonged lag time results in small colony formation in *Staphylococcus aureus* due to a slow growth rate.^36^ Stress-induced small colony formation has been identified in many species, such as *Salmonella enterica*, *P*. *aeruginosa*, and *E*. *coli*.^37^ Small colony variants carry memories of previous stress through reduced metabolic activity or *de novo* mutations in thymidine or hemin biosynthesis, resulting in defects in electron transport.^37^ It has been proposed that the high surface-to-volume ratio of cell sizes favors the control of intracellular drug concentrations (via reduced influx) and the binding of drugs to cell surfaces.^38^

The memory effect that causes reduced inhibition upon re-exposure to sublethal antibiotics is partially attributed to the aggregation of proteins that were formed during previous exposures and subsequently carried into daughter cells due to asymmetric division.^39^ Aggregated proteins confer a wide range of protective effects against different proteotoxic stresses, such as those imposed by ROS, drug exposure, and adverse environments.^40^ As an example, heat stress-induced proteins aggregate in *E*. *coli*, and offspring with inherited protein aggregates survived 20% more than those without when exposed to the same stress.^41^ In particular, functional protein aggregates, such as amyloids, are synthesized in the cytosol and then secreted through membrane permease to act as scaffold fibrils for biofilm production.^42^ Biofilms thus confer bacterial tolerance to a variety of stresses, including drugs. From the genotypic point of view, the carried-over effect could also be due to an inherited epigenetic shift, which will be discussed further as genotypic memory.

The response memory effect of increased survival upon re-exposure to lethal antibiotics is based on the enrichment of persisters from previous exposure.^35^ Tolerance mechanisms for persisters include decreased influx,^43^ increased efflux,^44^ enhanced DNA repair,^43^^,^^44^ and increased antioxidant capacity.^45^ For example, *E*. *coli*, which sustained 1 h of carbon starvation (a typical persister-inducing environment), increased the survival fraction 100-fold after 4 days of ciprofloxacin treatment.^46^ As a consequence, persisters have been identified as a clinical challenge often responsible for antibiotic treatment failure. Together, these results summarize memory effects associated with different phases or behaviors of bacterial responses and the underlying mechanisms; these results may help elucidated potential evolutionary interventions.

## Phenotypic and genotypic origins of bacterial memory

Commonly, history-dependent behaviors are classified as phenotypic traits, i.e., transcriptional memory, adaptive regulation during hysteresis, and memory-like responses after resuscitation, which are not inheritable. However, protein-based inheritance has been increasingly recognized. Specifically, protein aggregates that are remnants of past stresses can be stored primarily at one of the bacterial cell poles, so that only one daughter cell inherits then.^37^ Asymmetric aggregated proteins can be carried over for many generations, thus providing a survival advantage (e.g., high reproductive rate, fast recovery, and elevated tolerance capacity) in fluctuating environments.^47^ This phenomenon is also known as bacterial aging.^42^^,^^48^ As an example, previously surface-adapted cells exhibit fluctuations in their intracellular levels of adenosine 3′,5′-cyclic AMP and type IV pili regulons (responsible for early biofilm formation), which persist for many generations even after detachment, leading to planktonic forms.^49^

Regarding genotypic inheritance, epigenetic regulation has been regarded as a hidden driver of persister formation that compromises long-term (inheritable) memory (or epigenetic memory).^50^ Bacteria use DNA methylation patterns to transmit information about the phenotypic regulatory state of the parent cell to the offspring cell. Specifically, during rejuvenation, cells resume growth and division, and cells carrying dormancy-related memories (i.e., via epigenetic alterations) may be transmitted and retained in preparation for re-entering dormancy under successive stresses. As a result, the frequency of persisters increases with time during each round of stress, driving rapid evolutionary adaptation. For example, when *E*. *coli* cells were exposed to amikacin daily for 5 h followed by a 1-day recovery in fresh medium, the persister level increased approximately 300-fold after three rounds of treatment, thus showing multidrug tolerance.^51^

Notably, heterogeneous populations originating from hysteresis and regrowth can be either phenotypic or genotypic. In terms of phenotyping, populations exhibit a variety of metabolic activities and growth rates that structure the population based on the bet-hedging strategy. Regarding the genotypic basis, high persistence is pleiotropically associated with high mutation rates.^52^ For instance, *HipA* (high persister protein A) is an enzyme that halts translation, and mutations in *HipA* triggered an up to 1000-fold increase in the persistence of *E*. *coli*.^53^ Genes involved in DNA repair (e.g., *recA*, *recC*, *ruvA*, *uvrD*), global transcriptional regulators (e.g., *rpoS*, *fis*), and efflux pumps (e.g., *oppB*, *acrB*) are likely to mutate in persisters or to induce persistence.^54^ There is no doubt that cellular memory on both genotypic and phenotypic grounds can be unstable in the absence of subsequent selection pressure. However, further research is necessary to gain a deeper understanding of the mechanisms involved in the maintenance and accumulation of memory-like variations, which ultimately contribute to the establishment of stable and genetically distinct cell lines. This advanced knowledge is crucial for unraveling the complexities of evolutionary dynamics.

## Implications of the memory effect on drug tolerance/resistance

Previous exposure has a profound effect on the subsequent development of drug tolerance/resistance. Although all cells are able to survive high doses of the antibiotic, the tolerant population exhibits unaltered MICs relative to susceptible populations, whereas the resistant mutants have elevated MIC values. 1) Reduced metabolic activity at the lags decreases drug uptake. Tolerance by lag is well documented,^55^ and reduced metabolic activity (i.e., slowed growth and nongrowing) due to previous exposure is one of the decisive drivers of the emergence of antibiotic tolerance. For example, once the proton motive force was suppressed, the sensitivities of *E*. *coli* and *S*. *aureus* to aminoglycosides were significantly reduced owing to low drug penetration.^56^ 2) Diversification of populations with different metabolic activity during growth arrest has been shown to be advantageous in surviving antibiotic therapy. An interesting study by Fridman et al.^57^ demonstrates that stressed *E*. *coli* employ strategies to optimize lag time, that is, extend the single-cell lag time and shape the evolution of variability at the population level, which efficiently leads to antibiotic tolerance. 3) The high mutation rate of persisters facilitates the emergence of resistant mutants. It is well recognized that tolerance promotes the emergence of antibiotic-resistant mutants;^58^ for example, a single exposure to fluoroquinolones damages DNA in persisters and evokes an SOS response. Thereafter, induced error-prone DNA repair led to the mutagenesis of persisters during the postantibiotic recovery period, and there was a more than 300-fold increase in resistant mutants.^12^ 4) Previous exposure-induced genetic variation promotes the emergence of resistance upon the reoccurrence of stress. The emergence of high-level resistance through mutation is contingent on the genetic background, and thus, the nonspecific panmutation landscape induced by previous exposure expedites the genotypic evolution of resistance.^59^ For example, once re-exposed to high levels of rifampicin, an 8-fold increase in mutation frequency was observed for *E*. *coli* with a prior low-level exposure.^60^ Hence, understanding the genetic basis of antibiotic tolerance, persistence, and resistance mutations may provide more insightful information in understanding shifts in susceptibility during intermittent exposure and ultimately predicting evolution. 5) Collective tolerance at the population level is also crucial, either through the protective effects of self-aggregation and programmed cell death or through quorum sensing and shared public goods after regrowth.^61^ For example, the *mazEF* system of *E*. *coli* can be activated by amino acid starvation, DNA damage, and oxidative stress. Subsequently, under lethal drug therapy, *mazEF* induces programmed cell death in one subpopulation and releases beneficial products for the survival of the other cells.^62^

Heteroresistance may also occur due to heterogeneous subpopulations that evolve during lag or after recovery with reduced drug susceptibility compared to the main population. This population-related resistance may be unstable or stable, meaning that the resistant phenotype may or may not be able to revert to the susceptible phenotype in the absence of antibiotic selective pressure.^63^ In the event of stable heteroresistance, resistance mutations (e.g., single nucleotide polymorphisms, insertions, and deletions) are usually stable; these mutations are mainly involved in the efflux and/or influx of drugs. For example, heteroresistance to tigecycline in *S*. *enterica* has been attributed to multiple mutations in *RamR*, resulting in increased levels of *RamR* transcription factors and overexpression of the downstream *AcrAB-TolC* and *OqxAB* efflux pumps.^64^ The mechanistic interpretation for the instability of heteroresistance is unstable amplification of resistance genes or stable mutations that are associated with high fitness costs. As an example, amplification of the *lepB* gene, which encodes the target of arylomicin, has been identified in unstable heteroresistant *E*. *coli* subpopulations with a variety of copy numbers ranging from 1 to 50 in different subpopulations.^65^ However, due to their low frequency and transient nature, either type of heteroresistance is difficult to classify and poses a specific clinical concern, as they may increase in frequency during antibiotic therapy and cause treatment failure. Only a limited number of studies have explored the correlation between history-dependent behavior and the emergence of heteroresistance, as well as the potential benefits of utilizing bacterial memory to enhance drug effectiveness through the control of heteroresistance levels.

## Implications and potential applications of nanotechnology relevant to bacterial memory

### Historical nano-exposure and drug tolerance/resistance

**Figure 3:**
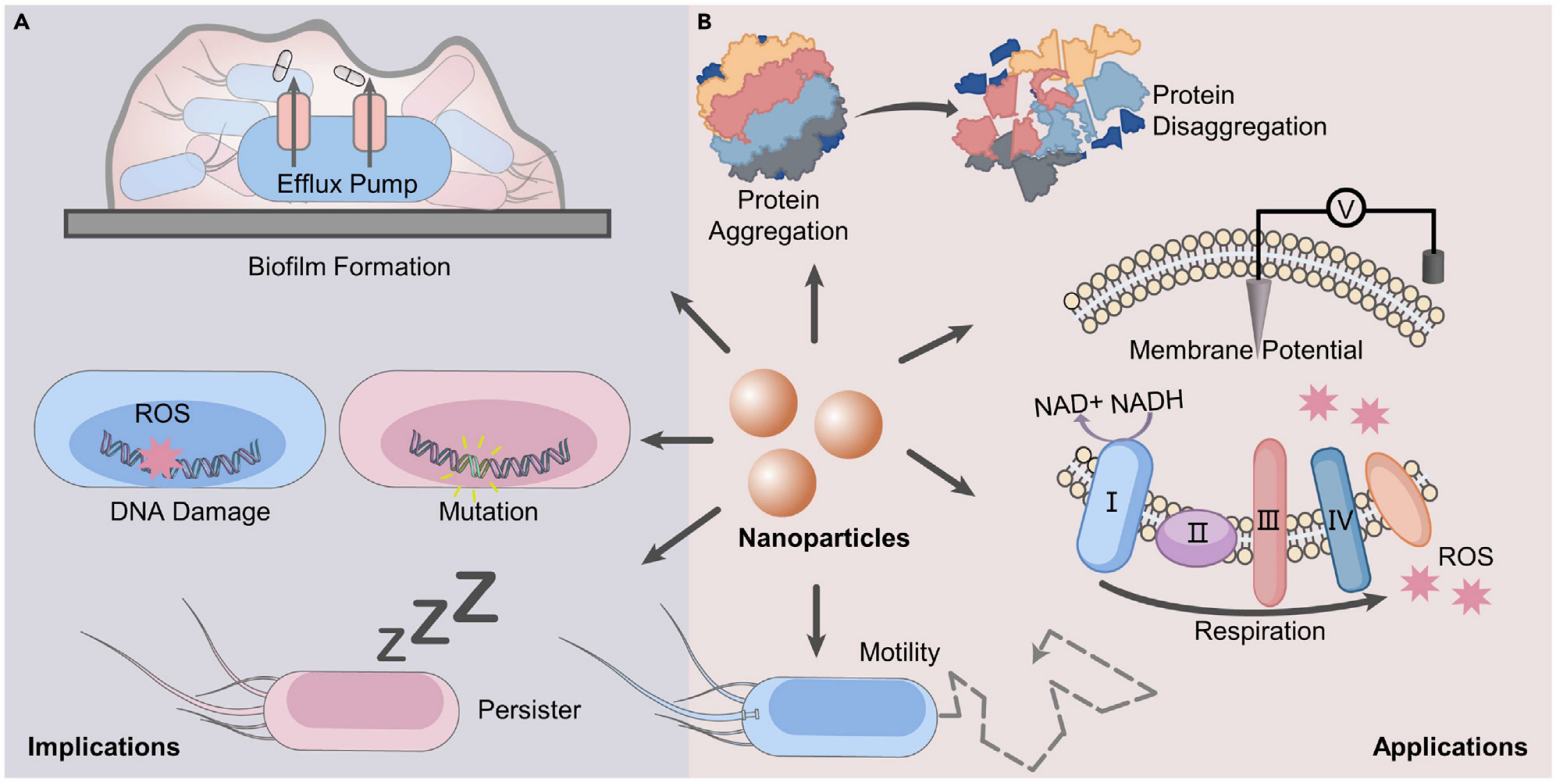
Nanoparticle exposure and memory effect (A) Nanoparticle-induced bacterial memory is implicated in drug tolerance/resistance. (B) Potential applications of nanotechnology that address the impacts of bacterial memory.

Sporadic information shows that nanoparticle-induced bacterial memory could be implicated in drug tolerance/resistance (Figure 3A); the results mainly showed 1) the induction and preservation of gene expression related to efflux pumps and biofilm formation. When preexposed to sublethal doses of silver nanoparticles, *E*. *coli* and *S*. *aureus* showed enhanced resistance to ampicillin, with IC_50_ (concentration that inhibits 50% bacterial growth) values elevated by a factor of 3–13.^66^ The upregulation of genes encoding metal efflux transporters and antioxidant systems is responsible for maintaining the intracellular redox balance and reducing the detrimental effects of drugs. 2) Nanoparticle exposure induces high mutagenesis driven by ROS, which subsequently evolves into drug resistance. For example, previous exposure of *E*. *coli* to 0.16–100 mg/L alumina oxide nanoparticles or 0.16–500 mg/L zinc oxide nanoparticles resulted in concentration-dependent changes in the frequency of resistance mutations under successive ciprofloxacin and chloramphenicol treatments. The nanoparticles significantly stimulated intracellular ROS production, leading to oxidative DNA damage and error-prone SOS responses, which corresponded to high mutagenesis in the subsequent drug treatment.^67^ 3) Antimicrobial nanoparticles may also induce high levels of persisters. Recently, a variety of metallic or metallic oxide nanoparticles have been shown to facilitate the formation of persisters by inducing hyperosmotic stress and the aggregation of the outer membrane proteins *ompA* and *ompC*.^68^ For instance, 13-, 5-, and 2-fold increases in persister numbers were detected upon treatment with copper nanoparticles, silver nanoparticles, and zinc oxide nanoparticles, respectively, at 200 μg/mL in conjunction with 150 μg/mL ampicillin. Notably, these mechanisms also contribute to the development of resistance against antimicrobial nanoparticles themselves, which could undermine the application of nanoparticles as an efficient weapon in the postantibiotic era.^69^ However, there is a knowledge gap regarding how bacteria gather information during each time window of nanoparticle exposure (i.e., bacterial memory), leading to antibiotic tolerance, which ultimately drives rapid and efficient evolution. This information is particularly imperative if one considers the use of nanotechnology to manipulate, suppress, or even reverse drug resistance by switching exposures.

### Mode of nanoparticle activity in manipulating bacterial memory

Very little information is available about using nanoparticles as “resistance breakers” to block certain bacterial resistance mechanisms; thus, the use of nanoparticles as adjuvants (commonly dosing without biocidal activity) brings a different dimension to antibacterial therapy that impacts the evolution of drug resistance. This information is highly essential considering the prevalence of resistance against β-lactam antibiotics and β-lactamase inhibitors. β-Lactamase inhibitors are adjuvants that are administered along with β-lactam antimicrobials to prevent resistance evolution by inhibiting serine β-lactamases. A recent study showed that 105 isolated strains from 150 patients admitted to the ICU were 100% resistant to either ampicillin-sulbactam or ampicillin-clavulanate.^70^ Therefore, employing distinctive, versatile, and tunable properties of nanoparticles beyond conventional chemical structures is a promising strategy. We tentatively summarize the potential structural properties of nanoparticles (Tabel 1) that differ from conventional chemicals and their applications in harnessing bacterial memory and intervention evolution (Figure 3B).

**Table 1:** Summarizes the promising features and functions of nanomaterials in harnessing bacterial memory

Mode of action	Features of nanomaterials	Type of nanomaterial	Reference
Disaggregate proteins	Chaperon-like activities	Metal nanoparticles, hydrophobic polymer nanoparticles, quantum dots, protein microspheres, and carbon nanoparticles	(Kogan et al.^71^; Yoo et al.^72^; Nag et al.^73^; Li et al.^74^; Singha et al.^75^; Pradhan et al.^76^; Hemeg^77^)
Tune membrane potential	Interactions with membranes, or block ion channel	Quantum dots, gold nanoparticles, nanoparticles with a positive surface, and single-wall carbon nanotubes	(Bloom et al.^78^; Nag et al.^79^; Dante et al.^80^; Park et al.^81^)
Interrupt microbial respiration	Electron transfer activity	Ti_3_C_2_T_x_ nanosheets, graphene oxide, carbon nanotubes, and fullerene	(Shen et al.^82^; Van den Bergh et al.^83^; Zhao et al.^84^; Vecitis et al.^85^; Zhang et al.^86^)
Regulate bacterial motility	Induction of flagella assembly or interaction with chemosensory via surface contact	Silver nanoparticles, graphene-based nanomaterials, and zinc oxide nanoparticles	(Stabryla et al.^87^; Zhang et al.^88^; Yan et al.^89^)

#### Disaggregate proteins

Nanoparticles with chaperone activity may activate persisters or prevent induction, which is believed to be an efficient tactic for resensitizing bacteria or avoiding tolerance. Dissolving protein aggregates associated with persistence are considered one of the first requirements for resuscitation, whereas chaperones play a key role in the prevention or reversal of protein aggregation. Various types of nanoparticles exhibit chaperone activity that inhibits protein aggregation, including metal nanoparticles,^71^ hydrophobic polymer nanoparticles,^72^ quantum dots,^73^ protein microspheres,^72^ and carbon nanoparticles.^74^ For instance, gold nanoparticles prevent the thermally induced aggregation of citrate synthase by affecting folding pathways.^75^ Iron oxide nanoparticles conjugated with glutamine and proline inhibit protein aggregation 1,000−10,000 times more efficiently than molecular glutamine and proline.^76^ The structural properties of nanoparticles responsible for chaperone activity have not been fully elucidated; thus, an insightful study of the structure-activity relation is necessary to provide more useful information in tuning nanostructures. Additionally, most of these studies were performed *in vitro* or in eukaryotic cells. Whether the nanoparticle size can accommodate the tiny size of the bacteria cell and how they can move into the cell or make contact with the cell envelope may also affect their chaperone-like activity. The application of nanoparticles as a potential treatment for the eradication of persisters via physical puncture or ROS generation has been previously reviewed^77^ and is not included here.

#### Tune membrane potential

Nanoparticles have also shown promise in tuning membrane potentials, which encode short-term memory and contribute to persister formation. Various nanoparticles, such as quantum dots and gold nanoparticles, excite/modulate the membrane potential by exploiting their optical properties.^78^ For example, a quantum dot-peptide-fullerene bioconjugate can be inserted into a membrane bilayer, with the hydrophilic quantum dot located outside the cell and the peptide-fullerene inserted into the membrane. The membrane potential can be depolarized with electron transfer from photoionized quantum dots to fullerenes.^73^ Additional modulation strategies involve electrostatic, mechanical interactions with membranes or the association of targeted ion channel proteins.^79^ For example, nanoparticles with a positive surface charge depolarize the membrane potential via an influx of calcium ions.^80^ Single-wall carbon nanotubes (0.9–1.3 nm in diameter and 1 μm in length) may block K^+^ currents in a dose- and size-dependent manner.^81^ Additional research is necessary from an application standpoint to gain a deeper understanding of the precise mechanism underlying the interaction between nanoparticles and cell membranes. These researches would aim to determine how such interactions can effectively modulate membrane potential and address the current knowledge gap regarding their subsequent influence on the preservation of cellular memory and the ability to adapt in the future.

#### Interrupt bacterial respiration

In addition, nanoparticles can interfere with microbial respiration, which is essential to supply energy for the detoxification or repair processes during lag. For example, Ti_3_C_2_T_x_ nanosheets, a typical MXene 2D nanomaterial, may stimulate bacterial respiration by targeting Complex I in the respiratory electron transport chain, leading to disequilibrium in NAD^+^ metabolism.^82^ The mechanism is coincident with L-serine, which is known to potentiate antibiotics against persisters by enhancing intracellular NADH and inducing ROS.^83^ This interruption of bacterial respiration has also been reported for other nanomaterials with high electron transfer activity, e.g., graphene,^84^ carbon nanotubes,^85^ and fullerene.^86^ As an example, graphene oxide interacts with the transmembrane protein cytochrome *c* and transfers electrons from the respiratory chain to extracellular oxygen, leading to the formation of extracellular ROS.^84^ Regarding bacterial memory, it is still unknown how such specific interventions shape evolutionary trajectories and what the evolutionary landscape looks like in the presence of nanomedicines.

#### Regulate bacterial motility

Finally, nanoparticles engage in motility-related regulation, which is essential for sensing nutrients, evading persistent states, and biofilm formation. Motility-related responses have been found in *E*. *coli* following exposure to silver nanoparticles rather than silver ions, with bacteria employing flagella assembly and the release of flagellins designed to precipitate nanoparticles.^87^ Our previous study showed that graphene-based nanomaterials may interact with chemosensors on bacterial surfaces to transitionally direct cell motion and simultaneously stimulate long-range swimming via the induced quorum sensing molecule AI-2.^88^ Another finding quantitatively revealed how the geometric surface arrangement and fine-tuning of the steric microenvironment of zinc oxide nanoparticles are capable of mediating the motility of *E*. *coli*.^89^ Research on motility-related regulation by nanoparticles is still in its early stages, and many questions remain unanswered. One important area of inquiry is how to effectively manipulate nanostructures in order to control bacterial motility behavior. Additionally, determining the essential transcription and translation factors involved in this process is of utmost importance. Another key aspect to consider is the interplay between nanoparticle-directed cell movement and the short-term memory response to drug stress. This interplay plays a significant role in long-term adaptation and the development of resistance. Understanding these complex dynamics will contribute to advancements in the field and pave the way for future applications.

## Conclusion and future perspectives

We summarized the history-dependent behavior in the dynamic phase of growth and related mechanisms and illustrate the origin of phenotypic and genotypic memory effects. Moreover, we demonstrated how preexisting regulatory networks influence bacterial susceptibility to drugs and explain how short-term bacterial memory is stored and involved in long-term adaptation and evolution. In particular, we searched regulatory networks derived from preexisting nanoparticle exposure to understand subsequent implications for drug tolerance/resistance. Finally, we paved the way for the potential application of nanotechnology in harnessing bacterial memory based on the unique properties of nanoparticles.

Understanding the memory effect and its underlying mechanism is of utmost importance for the application of nanotechnology in combating antibiotic resistance. On the one hand, this knowledge is crucial for the development of evolution-proof antimicrobial nanomaterials that prevent nanoresistance evolution.^90^ This becomes particularly critical when considering the widespread prevalence of resistance to silver nanoparticles among environmental microorganisms and clinically isolated pathogens.^91^ On the other hand, comprehending the decision-making process involved in bacterial memory formation allows us to explore the potential outcomes of combining nano-exposure with antibiotic therapy. By leveraging past events, we can guide future bacterial responses, ultimately halting the evolution of resistance. However, it should be noted that the application of nanomaterials to evolutionary biology is still in its early stages. Therefore, further systematic exploration is required in various aspects, such as exposure timing, ordering, transitional collateral effects, and structural influences.

First, memory-related regulatory networks are relatively short-lived, and the timing of a secondary stress (i.e., nanoparticle intervention) could have a decisive influence because the interactions between previous and ongoing gene networks may occur synergistically, antagonistically, or independently depending on the pattern of overlapping. As an illustration, a computational approach has been used to model the emergence of tolerance by lag in bacterial populations owing to antibiotic stress.^92^ The mathematical framework is able to predict phenotypic diversity and drug tolerance based on broad-tailed lags.^92^ A similar approach can be performed to assess historical exposure to nanomaterials, either alone, in combination, or sequentially with drugs. Additional computational simulations may be required to predict the outcome of a superposition of different responses based on the elapsed time of the prestimulated network, the functional connectivity with feedback, and the plasticity across gradients of the signaling molecules.

Second, the memory effect is strongly dependent on past experience; thus, the treatment sequence is imperative. Several studies have reported the effect of drug sequences on the evolutionary efficiency of bacterial tolerance/resistance;^93^ nevertheless, it is primarily unknown whether order-specific effects involving nanomaterials follow similar patterns as antibiotics or are more complex than thought, especially given the distinct structure and properties of nanomaterials. Our previous studies have shown that sequential treatment with ciprofloxacin and silver nanoparticles hinders the development of ciprofloxacin resistance by modulating the B12-dependent folate and methionine cycles, which cannot be predicted solely from the properties or biocidal mechanisms of silver nanoparticles.^94^ Therefore, further mechanistic studies of order-specific effects may help guide rational predictions of evolutionary dynamics and design nanotechnology-based strategies to mitigate resistance.

Third, transient collateral sensitivity related to the nanoparticle exposure history should be explored. Collateral sensitivity is an evolutionary trade-off that defines a situation in which a bacteria’s resistance to one drug may increase its susceptibility to other antibiotics.^95^ Mechanisms include resistance-related fitness costs and pleiotropic effects that potentiate high drug concentrations at the target site.^96^ In clinical trials, collateral sensitivity has been exploited to reduce the rate of resistance evolution by using drug pairs in combination or sequentially.^97^ As an example, dequalinium chloride has been used in *P*. *aeruginosa* to induce transient sensitivity to tobramycin, and subsequent exposure to tobramycin effectively kills cells, including those resistant to tobramycin.^98^ It would be fascinating to understand whether a temporary collateral sensitivity map could be produced in association with transient nanoparticle exposure. The results provide imperative information for the treatment of resistant pathogens and the design of evolutionary strategies to avoid the steady acquisition of antibiotic resistance mutations.

Finally, there is a knowledge gap regarding the relationship between the structural properties of nanomaterials and bacterial evolvability, which is essential for the application of nanotechnology as an evolutionary breaker. Current state-of-the-art approaches to antibiotic resistance breakers focus on the chemical structure of the inhibitor (e.g., β-lactamase inhibitor), which can interact with the drug enzyme to regain sensitivity.^99^ At the same time, searches for “evolution-proof antibiotics” have concentrated on bioactive compounds that target specific sites where sequences are highly conserved and few mutations can occur.^99^ Recently, an excellent review summarized proof-of-concept studies for the design of evolution-proof antimicrobial nanomaterials.^100^ The main mechanisms include extracellular attacks on the envelope and the constituents, employing multiple modes of action simultaneously and characteristic physical stresses.^100^ Evolutionary processes can thus be manipulated by diversifying microbial-nanoparticle interactions, controlling biocidal effects, and enhancing selectivity through structural modulation.^100^ Additional systematic studies on nano-bio interactions are needed to facilitate the rational design of nanostructures for various applications, including breakers and strategies resistant to evolution.

In summary, the exploration of bacterial memory and the understanding of its underlying mechanisms offer a promising opportunity to address the evolution of resistance and explore potential applications of nanotechnology in directed evolution.
